# Supplementary figures and images for: Validation of reference genes for gene expression studies in post-harvest leaves of tea plant (Camellia sinensis)
Source: PeerJ. 2019 Jan 31;7:e6385. doi: 10.7717/peerj.6385 (PMC6360078; doi:10.7717/peerj.6385)

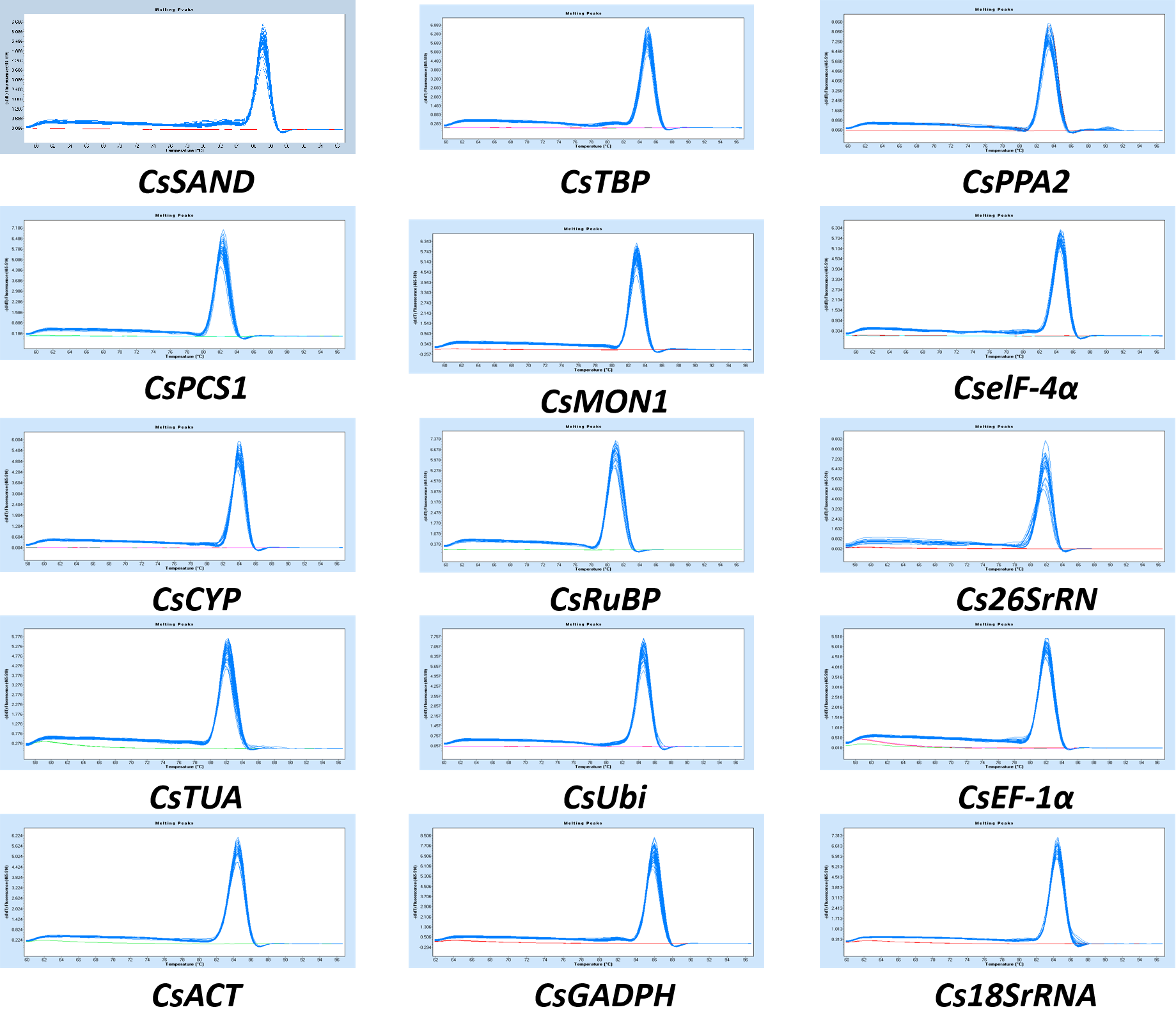

Supplement: Supplemental Information 1 [file peerj-07-6385-s001.png]
